# Diagnostic Effectiveness of [^123^I]Ioflupane Single Photon Emission Computed Tomography (SPECT) in Multiple System Atrophy

**DOI:** 10.3390/jcm12103478

**Published:** 2023-05-15

**Authors:** Javier Villena-Salinas, Simeón José Ortega-Lozano, Tomader Amrani-Raissouni, Eduardo Agüera, Javier Caballero-Villarraso

**Affiliations:** 1 Nuclear Medicine Service, Virgen de la Victoria University Hospital, 29010 Málaga, Spain; villenajavier@outlook.es (J.V.-S.); simeon.ortega.sspa@juntadeandalucia.es (S.J.O.-L.); tomader.amrani.sspa@juntadeandalucia.es (T.A.-R.); 2 Neurology Service, Reina Sofia University Hospital, 14004 Córdoba, Spain; doctoredu@gmail.com; 3 Maimónides Biomedical Research Institute of Córdoba (IMIBIC), 14004 Córdoba, Spain; 4 Clinical Analyses Service, Reina Sofía University Hospital, 14004 Córdoba, Spain; 5 Department of Biochemistry and Molecular Biology, Universidad of Córdoba, 14004 Córdoba, Spain

**Keywords:** multiple system atrophy, dysautonomia, functional neuroimaging testing, diagnostic accuracy, Ioflupane-123, cross-sectional study

## Abstract

Background: Multiple system atrophy (MSA) is a rapidly progressive neurodegenerative disorder that has no curative treatment. Diagnosis is based on a set of criteria established by Gilman (1998 and 2008) and recently updated by Wenning (2022). We aim to determine the effectiveness of [^123^I]Ioflupane SPECT in MSA, especially at the initial clinical suspicion. Methods: A cross-sectional study of patients at the initial clinical suspicion of MSA, referred for [^123^I]Ioflupane SPECT. Results: Overall, 139 patients (68 men, 71 women) were included, 104 being MSA-probable and 35 MSA-possible. MRI was normal in 89.2%, while SPECT was positive in 78.45%. SPECT showed high sensitivity (82.46%) and positive predictive value (86.24), reaching maximum sensitivity in MSA-P (97.26%). Significant differences were found when relating both SPECT assessments in the healthy–sick and inconclusive–sick groups. We also found an association when relating SPECT to the subtype (MSA-C or MSA-P), as well as to the presence of parkinsonian symptoms. Lateralization of striatal involvement was detected (left side). Conclusions: [^123^I]Ioflupane SPECT is a useful and reliable tool for diagnosing MSA, with good effectiveness and accuracy. Qualitative assessment shows a clear superiority when distinguishing between the healthy–sick categories, as well as between the parkinsonian (MSA-P) and cerebellar (MSA-C) subtypes at initial clinical suspicion.

## 1. Introduction

Multiple system atrophy (MSA) is a progressive degenerative process of the central and autonomic nervous systems. It usually happens in adulthood, with sporadic incidence. It causes severe disability in the medium term and leads to death in less than 10 years [1,2]. The disease affects (variably and in any combination) the nigrostriatal, olivopontocerebellar, autonomic, and corticospinal systems, along with a lack of response to levodopa treatment [1]. The involvement of the nigrostriatal neuronal pathway develops the parkinsonian syndrome consisting of tremors, bradykinesia/rigidity and postural instability [3].

The diagnosis of MSA is based on widely accepted criteria that classify the disease as possible, probable or definite (depending on the patient’s symptoms) [4]. These criteria were reviewed in 2008, including neuroimaging tests [5]. One of them was brain SPECT with presynaptic dopamine transporters, which was included for possible MSA type C until the latest revision of the diagnostic criteria [6].

This SPECT allows detecting “in vivo” changes at the molecular level that affect brain dopaminergic function, specifically the nigrostriatal pathway. One of the most widely used radiopharmaceuticals is [^123^I]Ioflupane, which makes it possible to visualize the density of presynaptic dopamine transporters in both striatal nuclei. The interpretation of this study consists of a qualitative assessment, where the uptake and distribution of the radiopharmaceutical in the striated nuclei are visualized. This visual analysis is sufficient for diagnosis. On the other hand, numerous image analysis software tools have emerged, which could help nuclear medicine physicians. However, there is currently no clear standardization in this respect and each company has its own quantification software.

The validity and usefulness of SPECT in the diagnosis of Parkinson’s disease (PD), as well as in clinically uncertain parkinsonian syndromes, even in early stages, has so far been demonstrated [7,8,9]. The main indications for SPECT are: (i) differential diagnosis between neurodegenerative parkinsonian syndrome and essential tremor; (ii) distinguishing between Lewy body dementia and other dementias such as Alzheimer’s disease; (iii) differential diagnosis between parkinsonism with presynaptic dopamine deficit (such as PD) and secondary parkinsonism (psychogenic, drug-induced, or vascular); and (iv) early detection of parkinsonian syndromes with presynaptic involvement [10].

It has not been demonstrated that it is possible to reliably discriminate between PD and MSA by SPECT. There are a multitude of studies that address this question, seeking both qualitative and quantitative differences, with varying results [11,12,13,14]. The greatest diagnostic difficulty is found when analyzing the subtypes of MSA: parkinsonian (MSA-P) and cerebellar (MSA-C) and the usefulness of [^123^I]Ioflupane SPECT. There is a certain consensus on its usefulness in MSA-P, being more questioned in the MSA-C subtype, given that, at the onset of the disease, SPECT can be completely normal [15,16,17]. For this reason, a negative result does not exclude the diagnosis of MSA [18]. In addition, it has been proposed to monitor the evolution of MSA by performing [^123^I]Ioflupane SPECT. In fact, it is postulated that this test could be an important biomarker of disease progression, as well as the subtype of dopaminergic degeneration [16,19].

The aim of this work is to determine the diagnostic effectiveness of brain [^123^I]Ioflupane SPECT in both subtypes of MSA, while addressing all the aforementioned controversies and lack of consensus. Possible differences between quantitative and qualitative analyses of this technique will also be analyzed.

## 2. Materials and Methods

### 2.1. Study Design and Subjects

A single-center observational cross-sectional study. Patients over 18 years of age, with clinical suspicion compatible with MSA according to Gilman’s criteria (probable/possible/definite), who had undergone [^123^I]Ioflupane SPECT during the period 2004–2020, were included. All DAT-SPECT scans were requested by the Movement Disorders Unit of the Neurology Department of our hospital, after the initial interview with the patient. All of them had to be able to understand the diagnostic procedure and give informed consent. All patients who did not meet these requirements were excluded, as well as those who showed signs of “red flags” [20] or were taking medication that could interfere with the SPECT result (qualitative and quantitative).

Demographic and clinical variables were obtained by means of a clinical interview conducted by the neurologist of the movement disorders unit, prior to SPECT. The mean time from clinical onset to neurologist visit was 5–9 days (mean 1 week). The mean time from onset from neurologist requesting SPECT to SPECT was 2 weeks (mean 1–3 weeks).

### 2.2. SPECT of Presynaptic Dopamine Transporters with [^123^I]Ioflupane

The SPECT tomographic study was performed with a Siemens Healthineers^®^ gamma camera, (Erlangen, Germany) model Symbia^TM^, equipped with a double head and a low energy and high resolution collimator. The images were obtained after a period of 3 to 4 h of intravenous administration of 185 MBq of the radiotracer [^123^I]Ioflupane, after thyroid blockade with lugol solution.

A 360° circular orbit around the skull was performed, with 3° intervals, acquiring 60 images with a duration of 35 s per interval, and a 128 × 128 matrix. Images were reconstructed using filtered back projection algorithms without attenuation correction, with the application of a Hanning filter (frequency of 0.7), and images were obtained according to transaxial slices and orbito-meatal orientation.

This study was first evaluated by qualitative analysis by three nuclear medicine physicians, two of them with extensive experience in the field of movement disorders. The type of MSA and its diagnostic classification (possible/probable) were blinded. A kappa index of 100% was obtained. The studies were also evaluated by semi-quantitative analysis, establishing uptake indices (after the sum of the six most representative axial images) between regions of interest (ROI) of an area of specific activity (striatal nuclei) and an area of non-specific activity (occipital cortex), obtaining the striatal/occipital index (S/O) [21]. This analysis can be performed independently of the gamma camera used and is highly reproducible.

### 2.3. Convectional MRI

Conventional MRI was performed in all patients. The MRI protocol performed included the following sequences: sagittal T1, axial T2, FLAIR, diffusion and echogradient. Contrast was not used. In Parkinson’s disease there are no findings, or they are non-specific, so MRI is performed to carry out the differential diagnosis with other parkinsonisms that can show neuroimaging findings.

### 2.4. Ethical Considerations

Authorization for this study was obtained from the Biomedical Research Ethics Committee of the province of Malaga. At all times, the harmonized tripartite standards of the Helsinki declaration, the Organic Law on Biomedical Research of 15/1999 of 3 July, the Organic Law on Personal Data Protection (LOPD) of 13 December 2018, the code of ethics of the Organización Médica Colegial (OMC), the basic regulatory law 41/2002 on patient autonomy and rights and obligations regarding clinical information and documentation, of 14 November, as well as the standards of good clinical practice, were respected.

### 2.5. Statistical Study

First, a descriptive study was performed, showing absolute and percentage frequencies for qualitative variables. Continuous quantitative variables were expressed as mean and standard deviation. The Shapiro–Wilk test was used to check whether the values of these variables followed a normal distribution.

To calculate the association between qualitative variables, the Chi-square test was applied with Fisher’s correction if appropriate. To analyze the differences between continuous quantitative variables, the Student *t* test (parametric) or Mann–Whitney U test (nonparametric) was used for two independent groups, and the ANOVA test (parametric) or Kruskal–Wallis test (nonparametric) in three or more independent groups. ROC curves were performed for different cut-off points according to quantitative SPECT analysis. A significance level was considered for *p* < 0.05. Confidence intervals were established at 95%.

## 3. Results

A total of 139 patients were studied: 68 males (48.9%) and 71 females (51.1%). The mean age was 68 years, with minimum and maximum ages of 47 and 85 years, respectively, and a standard deviation of 9. 104 cases were classified according to clinical criteria as MSA-probable (74.8%), 35 as MSA-possible (25.2%), and no subject was found with MSA-definite. The predominant subtype was MSA-P (62.6%) versus MSA-C (37.4%). Of the patients, 84.2% had parkinsonian syndrome, 40.3% had cerebellar symptomatology, 62.6% had dysautonomia and 20.1% had corticospinal symptoms. Some 94.2% showed no response to levodopa treatment, while the remaining 5.8% had a transient response.

Convectional MRI was anodyne in 89.2% of patients. The SPECT study with [^123^I]Ioflupane was compatible with nigrostriatal pathway involvement in 78.45%, with a non-pathological study in 21.55% (16.6% being normal and 5% inconclusive). The mean scores of the uptake indices obtained by SPECT were 1.35 ± 1.74 (0.9–1.88) for the global S/O index, 1.36 ± 1.8 (0.9–1.86) for the right S/O index and 1.34 ± 1.7 (0.91–1.89) for the left S/O.

### 3.1. Diagnostic Validity Study

Accuracy values were calculated for the entire MSA caseload (139 patients) and for both types of the disease: MSA-P (87 patients) and MSA-C (52 patients), shown in Table 1.

### 3.2. Qualitative vs. Quantitative SPECT Categorization

It is observed that there are significant differences in the scores of the three S/O indexes analyzed (global, right and left), in two of the groups defined by the qualitative assessment made by the nuclear medicine physician: pathological vs. inconclusive group (*p* < 0.05) and pathological vs. normal group (*p* < 0.001), as shown in Table 2.

### 3.3. Relationship between SPECT Categorizations (Qualitative and Quantitative) and Clinical Diagnosis (Gilman’s Criteria, 2008)

We analyzed whether there are significant differences between the scores of the three quantitative indices, as well as between the three groups defined by visual assessment, according to the diagnostic classification (probable MSA, possible MSA), but no significant differences were obtained.

### 3.4. Cut-Off Proposition for the Overall S/O Index in Relation to the Qualitative Categorization Performed by the Nuclear Medicine Physician

**Figure 1:**
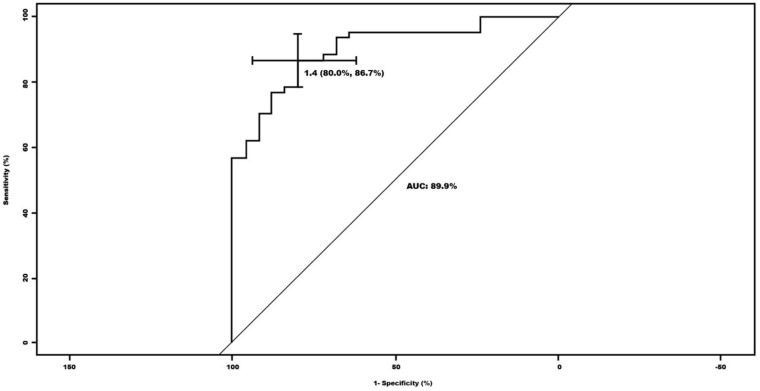
ROC curve for the global striatum/occipital (S/O) index in relation to the qualitative categorization of the nuclear medicine physicians for the normal versus pathological groups.

The optimal cut-off for the global S/O index in relation to the qualitative categorization for the normal vs. pathological groups was established at 1.4. Based on this, a sensitivity of 80% and a specificity of 86.7% were obtained. The area under the curve (AUC) was 89.9%, and the range was 83.3–96.5% (Figure 1).

### 3.5. Cut-Off Proposition for the Global S/O Index in Relation to Clinical Diagnosis

**Figure 2:**
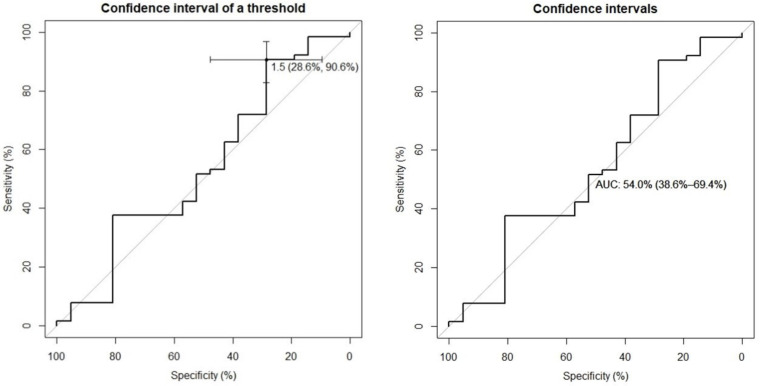
ROC curves for the global striatum/occipital (S/O) index in relation to the clinical diagnosis, for the normal versus pathological groups.

When comparing this index with the diagnostic classification made by the neurologist (Gilman’s criteria) in both groups mentioned (normal vs. pathological), an optimal cut-off of 1.5 was obtained, according to which we have a sensitivity of 28.6% and a specificity of 90.6%. The area under the ROC curve (AUC) was 54%, and the range was 38.6–69.4% (Figure 2).

### 3.6. Relationship between MSA Subtypes, SPECT Categorization (Qualitative and Quantitative) and Clinical Diagnosis

Significant differences were found between MSA subtype (MSA-C or MSA-P) and both SPECT categorizations (both with *p* < 0.01), but not so with clinical diagnosis (*p* = 0.11) (Table 3).

### 3.7. Lateralization in Striatal Involvement of MSA

We studied whether one cerebral hemisphere is more affected than the other at the onset or during the development of the disease and whether this preponderance has an impact on other variables. In this regard, according to the quantitative SPECT assessment, we observed that there are significant differences between the mean values of the striatal nuclei of both cerebral hemispheres, obtained from the right S/O index and left S/O index (mean 1.4 (SD 0.2) vs. mean 1.3 (SD 0.2), *p* < 0.05). Similarly, according to the visual assessment performed by the nuclear medicine physician, it was observed that 57.65% of the patients had greater involvement of the left striate nucleus.

When trying to find associations between the predominant hemisphere with other variables (qualitative SPECT assessment, MSA subtype and diagnostic classification), no significant differences were obtained (Table 4).

### 3.8. Response to Levodopa

We analyzed the responsiveness to levodopa in MSA as a diagnostic resource and compared it with other diagnostic or classification strategies. We found no association between the response to this treatment and the different variables used (diagnostic classification, SPECT categorizations and type of MSA) (Table 5).

### 3.9. Relationship between Parkinsonian Clinic, SPECT Findings and MSA Subtype

Significant differences were found between the presence of clinical parkinsonism and the SPECT result (for both qualitative and quantitative assessments), as well as with the MSA subtype (MSA-C or MSA-P). Significant differences were found in all of them, except for clinical diagnosis (Table 6).

## 4. Discussion

According to our study, we can observe a high reliability of the diagnosis issued by the nuclear medicine physician at initial clinical suspicion of MSA. This is demonstrated when we analyze the relationships between the visual (qualitative) diagnostic classification of [^123^I]Ioflupane SPECT and quantitative assessment (with predefined ROIs method). There is a parallelism between this visual assessment and the quantitative value of SPECT. Currently there is a multitude of specific software to quantitatively assess the SPECT image, but there is no diagnostic standardization. This fact, in conjunction with the results of our study, allows us to propose our semi-quantification method because of its simplicity and high reproducibility.

The SPECT result (qualitative and quantitative) was related to the MSA subtype. This is to be expected from a pathophysiological point of view, given that SPECT detects nigrostriatal pathway involvement even in the prodromal phase of the disease [7,8]. This affectation is the origin of the parkinsonian clinic (which is mostly present in the MSA-P form) [1,2]. In any case, it should be noted that SPECT abnormalities in a patient with parkinsonism are not specific of MSA.

However, there was no relationship between the presence of parkinsonian symptoms and the diagnostic classification of the disease (probable or possible), the closest to a true MSA without postmortem confirmatory study (according to diagnostic criteria) [5]. Nevertheless, the previous clinical diagnostic classification (probable/possible) does not fully correspond with the SPECT assessment (both qualitative and quantitative), despite the fact that most of our patients (74%) were probable MSA [4,5]. Additionally, for cases not confirmed by autopsy (possible MSA/probable MSA), [^123^I]Ioflupane SPECT can be an accurate and non-invasive in vivo study.

Since there is still no clear consensus within quantitative assessment to establish a cut-off to consider a study normal or pathological, we propose, based on our results (probably the first study with such a large number of patients), that this cut-off can be set at 1.4, since it provides good values of sensitivity (80%) and specificity (86%).

On the other hand, we have not been able to establish a useful cut-off in the diagnostic classification (probable/possible). For a cut-off of 1.5, we obtained a good specificity (90.6%), although with a low sensitivity and area under the curve (28.6% and 50.4%, respectively). Therefore, it does not appear that clinical classification has an impact on the SPECT quantitative assessment.

In the present study, the proportion of patients with MSA was similar in men and women, which is consistent with the scientific literature [22]. However, initially, a higher percentage of males may present for consultation due to the clinical manifestations of erectile dysfunction [23].

The age distribution of the patients is also consistent with the literature [3,24,25,26,27]. It should be added that the age of our patients does not refer to the clinical onset, but to the timing of SPECT, which is usually requested after a first or second consultation in the movement disorders unit. The time elapsed between the clinical debut and the performance of the imaging test could be of importance in older subjects, as it could be due to a delay in clinical suspicion. We recommend further studies with this subgroup of patients, as it would be interesting to analyze their clinical particularities (coexistence of multiple signs and symptoms, other concomitant pathologies, greater difficulty in using current diagnostic criteria, etc.) [20]. Such particularities may mean that these elderly patients with suspected MSA require earlier care and/or closer follow-up.

The most frequent subtype of MSA was MSA-P, with a 3:1 ratio with respect to MSA-C. These data coincide with those published by the European MSA study group (EMSA group) where, although a higher percentage of MSA-C was obtained in Spain, the overall results show a predominance of MSA-P in Europe, with a homogeneous distribution [26,28,29,30].

In this aspect, [^123^I]Ioflupane SPECT is useful to diagnose both subtypes, since we found significant differences for both qualitative and quantitative assessment. This finding is interesting, since the vast majority of our patients had parkinsonian syndrome and less than half of them had cerebellar symptomatology. Another important element seems to be the manifestation of dysautonomia, also very frequent in our patients, but not the presence of corticospinal symptoms. These results are in agreement with the diagnostic criteria regarding the clinical characteristics of this disease and their impact on establishing the diagnosis [5,6].

The scarce or null response to levodopa treatment in practically all of our patients coincides with that previously described by other authors [1,31]. In this context and given that there does not seem to be a clear association between the response to levodopa and the SPECT result (qualitative or quantitative), we questioned whether, in the presence of a pathological SPECT result in suspected MSA, we could dispense with this drug. This would imply a reduction in costs and would avoid potential adverse effects for patients [32,33].

Similarly, conventional MRI did not prove useful in the diagnosis of MSA in the majority of patients (89.2%). Since SPECT is performed at the same time as MRI upon initial suspicion of MSA, perhaps its usefulness lies in the detection of other pathologies that could be confused with MSA. It has been reported that this imaging technique usually shows nonspecific signs in atypical parkinsonism, with structural alterations appearing when the disease is advanced [34,35].

In contrast, [^123^I]Ioflupane SPECT was positive in most patients (78.45%). This is similar to previously published studies, given that there may be integrity of the nigrostriatal pathway in early cases of MSA, especially in the MSA-C subtype [17]. In this context, it would be interesting to perform an evolutionary study in this subgroup of patients, since the rapidly progressive deterioration of the disease could confirm or rule out the existence of MSA at a later stage. In the literature, we found hardly any research on this aspect [16,19,36,37].

We can confirm the high sensitivity of SPECT for the diagnosis of MSA (82%), especially in patients with MSA-P (97%) with respect to MSA-C. On the other hand, it has a high positive predictive value (PPV > 85%) in both subtypes, so we believe that in the clinical suspicion of MSA, SPECT is a very useful tool. These results correspond to those of other authors [9]. This tool could be considered for those incipient cases, perhaps even as diagnostic support in the prodromal phases (as the new category published) [6]. Furthermore, it is a widely available technique in our setting compared to others, such as PET-CT, for example, which is not available in all hospitals. For all these reasons, we propose that the role of [^123^I]Ioflupane SPECT can be considered in the current diagnostic criteria.

Quantitative SPECT analysis showed that both striatal nuclei were significantly impaired, being more frequent the initial involvement of the left striatal nucleus. This lateralization has not been previously described, so we believe it is a finding that should be taken into account when diagnosing the disease. Moreover, it could be a distinctive feature to differentiate MSA from other entities. Therefore, it would be interesting to know how this condition evolves, by means of follow-up studies. In this regard, a baseline study could be performed (either at the onset of the disease or at any stage of the disease), in order to corroborate this deterioration at a later time. Previous publications point out the usefulness of [^123^I]Ioflupane SPECT to monitor the evolution of this disease [16,19].

## 5. Conclusions

In conclusion, we could state: (i) [^123^I]Ioflupane SPECT is a useful and reliable tool in the diagnosis of MSA (especially upon initial suspicion), with higher sensitivity and accuracy than other conventional imaging techniques. (ii) The qualitative assessment shows a net superiority in discerning between healthy–sick categories, as well as between MSA-P and MSA-C subtypes, which denotes that the assessment of the nuclear medicine physician has a relevant point in the diagnosis. There is also a good correspondence between the nuclear medicine physician’s assessment and the quantitative assessment of [^123^I]Ioflupane SPECT using predefined ROIs and our proposed cut-off. (iii) Treatment with levodopa does not seem to provide benefits in the diagnosis of patients with MSA, so in the presence of a positive [^123^I]Ioflupane SPECT study, we believe that this drug could be omitted in terms of cost-effectiveness and cost-efficiency. (iv) Most of our patients showed an initial predominant involvement of the left striatal nucleus. This finding could be interesting for its possible usefulness in the diagnosis of MSA, especially to identify incipient cases of this disease.

## Figures and Tables

**Table 1:** Diagnostic accuracy values.

	MSA	MSA-P	MSA-C
N	139	87	52
S	82.46% (75.04–89.88)	97.26% (92.83–100.00)	56.10% (39.69–72.51)
E	40% (18.80–61.20)	21.43% (0.00–46.49)	63.64% (30.66–96.61)
PPV	86.24% (79.31–93.16)	86.59% (78.60–94.57)	85.19% (69.93–100.00)
PNV	33.33% (14.80–51.87)	60.00% (7.06–100.00)	28% (8.40–47.60)
FP	60%	79%	36%
FN	18%	3%	44%
Accuracy	10%	85%	58%

MSA: Multiple System Atrophy; MSA-P: Multiple System Atrophy Parkinsonian Type; MSA-C: Multiple System Atrophy Cerebellar Type; N: Number of patients; S: Sensitivity; E: Specificity; PPV: Positive predictive value; PNV: Negative predictive value; FP: False positives; FN: False negatives. The 95% confidence interval is shown in parentheses.

**Table 2:** Analysis of relations between qualitative and quantitative SPECT categorizations.

S/O Indices	Groups			*p*-Value
	Pathological	Normal	Inconclusive	
Global	1.279 (0.135) ^ἠ^	1.522 (0.155)	1.467 (0.064)	<0.001 ***
Right	1.287 (0.142) ^ἠ^	1.530 (0.161)	1.478 (0.077)	<0.001 ***
Left	1.271 (0.136) ^ἠ^	1.515 (0.152)	1.455 (0.057)	<0.001 ***

S/O: Striatum/Occipital; ^ἠ^ *p* < 0.05 pathological vs. inconclusive; *** *p* < 0.001 pathological vs. normal.

**Table 3:** Relations between MSA subtypes, SPECT categorizations and diagnosis.

Variables		MSA Type		*p* Value
		AMS-C	AMS-P	
Diagnosis	Probable	43 (82.7%)	61 (70.1%)	*p* > 0.05
	Possible	9 (17.3%)	26 (29.9%)	
SPECT qualitative assessment	Pathological	27 (51.9%)	82 (94.2%)	***
	Normal	20 (38.5%)	3 (3.4%)	
	Inconclusive	5 (9.6%)	2 (2.3%)	
SPECT quantitative assessment	Global S/O	1.452 (0.177)	1.271 (0.128)	***
	Right S/O	1.459 (0.185)	1.280 (0.133)	***
	Left S/O	1.445 (0.172)	1.262 (0.130)	***

S/O: Striatum/Occipital; MSA: Multiple System Atrophy; MSA-P: Multiple System Atrophy Parkinsonian Type; MSA-C: Multiple System Atrophy Cerebellar Type; *** *p* < 0.001.

**Table 4:** Relations between the dominant hemisphere and other variables.

Variables		Cerebral Dominance		*p* Value
		Right	Left	
SPECT qualitative assessment	Pathological	34 (69.4%)	26 (72.2%)	*p* > 0.05
	Non-pathological	15 (30.6%)	10 (27.8%)	
MSA type	MSA-C	19 (38.8%)	17 (47.2%)	*p* > 0.05
	MSA-P	30 (61.2%)	19 (52.8%)	
Clinical diagnosis	MSA-possible	12 (24.5%)	9 (25%)	*p* > 0.05
	MSA-probable	37 (75.5%)	27 (75%)	

MSA: Multiple System Atrophy; MSA-P: Multiple System Atrophy Parkinsonian Type; MSA-C: Multiple System Atrophy Cerebellar Type.

**Table 5:** Relations between response to levodopa treatment and other variables.

Variables		Response to Levodopa		*p* Value
		NO	YES	
Diagnosis	Probable	97 (74%)	7 (87.5%)	*p* > 0.05
	Possible	34 (25.9%)	1 (12.5%)	
SPECT qualitative assessment	Pathological	104 (79.4%)	5 (62.5%)	*p* > 0.05
	Non-pathological	27 (20.6%)	3 (37.5%)	
SPECT quantitative assessment	Global S/O	1.345 (0.175)	1.407 (0.174)	*p* > 0.05
	Right S/O	1.352 (0.180)	1.424 (0.188)	*p* > 0.05
	Left S/O	1.337 (0.175)	1.390 (0.162)	*p* > 0.05
MSA type	MSA-C	49 (37.4%)	3 (37.5%)	*p* > 0.05
	MSA-P	82 (62.6%)	5 (62.5%)	

S/O: Striatum/Occipital; MSA: Multiple System Atrophy; MSA-P: Multiple System Atrophy Parkinsonian Type; MSA-C: Multiple System Atrophy Cerebellar Type.

**Table 6:** Relations between presence of parkinsonian symptoms and other variables.

Variables		Parkinsonian Symptoms		*p* Value
		NO	YES	
Diagnosis	Probable	15 (68.2%)	89 (76.1%)	*p* > 0.05
	Possible	7 (31.8%)	28 (23.9%)	
SPECT qualitative assessment	Pathological	9 (40.9%)	100 (85.5%)	***
	Non-pathological	13 (59.1%)	17 (14.5%)	
SPECT quantitative assessment	Global S/O	1.522 (0.153)	1.301 (0.149)	***
	Right S/O	1.529 (0.162)	1.309 (0.155)	***
	Left S/O	1.515 (0.147)	1.293 (0.150)	***
MSA type	MSA-C	22 (100%)	30 (25.6%)	***
	MSA-P	0	87 (74.4%)	

S/O: Striatum/Occipital; MSA: Multiple System Atrophy; MSA-P: Multiple System Atrophy Parkinsonian Type; MSA-C: Multiple System Atrophy Cerebellar Type; *** *p* < 0.001.

## Data Availability

Not applicable.
